# Transcriptional profiling of mycobacterial antigen-induced responses in infants vaccinated with BCG at birth

**DOI:** 10.1186/1755-8794-2-10

**Published:** 2009-02-24

**Authors:** Helen A Fletcher, Alana Keyser, Mark Bowmaker, Peter C Sayles, Gilla Kaplan, Greg Hussey, Adrian VS Hill, Willem A Hanekom

**Affiliations:** 1 Jenner Institute, ORCRB, University of Oxford, Churchill Hospital, Oxford, OX3 7DQ, UK; 2 South African Tuberculosis Vaccine Initiative, Institute of Infectious Diseases and Molecular Medicine, and School of Child and Adolescent Health, University of Cape Town, South Africa; 3 Trudeau Institute Inc., Saranac Lake, NY, USA; 4 Laboratory of Mycobacterial Immunity and Pathogenesis, Public Health Research Institute, Newark, NJ, USA

## Abstract

**Background:**

Novel tuberculosis (TB) vaccines recently tested in humans have been designed to boost immunity induced by the current vaccine, *Mycobacterium bovis *Bacille Calmette-Guérin (BCG). Because BCG vaccination is used extensively in infants, this population group is likely to be the first in which efficacy trials of new vaccines will be conducted. However, our understanding of the complexity of immunity to BCG in infants is inadequate, making interpretation of vaccine-induced immune responses difficult.

**Methods:**

To better understand BCG-induced immunity, we performed gene expression profiling in five 10-week old infants routinely vaccinated with BCG at birth. RNA was extracted from 12 hour BCG-stimulated or purified protein derivative of tuberculin (PPD)-stimulated PBMC, isolated from neonatal blood collected 10 weeks after vaccination. RNA was hybridised to the Sentrix^® ^HumanRef-8 Expression BeadChip (Illumina) to measure expression of >16,000 genes.

**Results:**

We found that ex vivo stimulation of PBMC with PPD and BCG induced largely similar gene expression profiles, except that BCG induced greater macrophage activation. The peroxisome proliferator-activated receptor (PPAR) signaling pathway, including PPAR-γ, involved in activation of the alternative, anti-inflammatory macrophage response was down-regulated following stimulation with both antigens. In contrast, up-regulation of genes associated with the classic, pro-inflammatory macrophage response was noted. Further analysis revealed a decrease in the expression of cell adhesion molecules (CAMs), including integrin alpha M (ITGAM), which is known to be important for entry of mycobacteria into the macrophage. Interestingly, more leukocyte genes were down-regulated than up-regulated.

**Conclusion:**

Our results suggest that a combination of suppressed and up-regulated genes may be key in determining development of protective immunity to TB induced by vaccination with BCG.

## Background

World-wide, two million people die from tuberculosis (TB) every year, and an estimated two billion people, a third of the world's population, are latently infected with *Mycobacterium tuberculosis *(*M.tb)*. TB is the leading identifiable cause of death among HIV-infected people [1]: an estimated quarter of a million deaths in HIV-infected persons per year are TB-associated. An improved vaccine against TB would be the most effective intervention for disease control. Bacille Calmette-Guérin (BCG), first used as a human vaccine in 1921, is one of the most widely administered vaccines in the world. BCG affords 80% protection against severe infant TB; however, protection against lung TB is variable and mostly poor, in all ages [2]. There is therefore an urgent need to develop improved TB vaccines. Multiple novel vaccine candidates which demonstrate some protection in mice challenged with virulent *M.tb *have emerged [3]. Most novel vaccine candidates are designed to boost immunity that was primed by prior BCG vaccination; however, not enough is known about immunity induced by BCG in adults. Even less is known about immunity in vaccinated *neonates*. This study attempted to address some of the gaps in our knowledge, by exploring mRNA expression profiles following BCG vaccination of newborns.

DNA microarrays are increasingly being used to assess mRNA expression profiles associated with host-pathogen interactions. In the TB research field, microarrays have been used to explore changes in gene expression in TB infected macrophages [4,5]. Also, differences in host responses between tuberculoid and lepromatous leprosy patients [6], and between patients with pulmonary and disseminated TB, have been studied [7]. Overall, reports of applications of arrays in infants are scanty, and include an assessment of differences in transcriptional profiling between acute and convalescent infant influenza infection, as a tool to discriminate acute infection states in infants [8,9]. Moreover, microarray analysis of infant PBMC has not been used in tuberculosis research, perhaps due to the large volumes of blood typically required for array analysis. PBMC are increasingly being used as a surrogate tissue for the molecular diagnosis of diseases involving less accessible tissues such as lung, kidney and heart. This is because changes in PBMC probably reflect pathological and immunological changes that occur elsewhere in the body [10]. Similarly, whole PBMC populations have been found useful for the assessment of differences in transcriptional responses induced by diverse mycobacterial antigens [7,11]. Here, we describe the development of a gene expression assay for the detection of antigen-specific responses in the PBMC of BCG vaccinated infants. Our aim was to assess specific differences in mRNA expression profiles induced by 2 mycobacterial antigens, *M. bovis *BCG and *M.tb *purified protein derivative of tuberculin (PPD), to guide antigen use in future, more detailed studies. The former antigen consists of a whole, viable avirulent mycobacterium, which can be phagocytosed and processed by monocytes in PBMC to present both protein and non-protein antigens. By contrast, PPD consists of only soluble proteins from virulent *M.tb*.

## Methods

### Study participants and blood collection

Healthy 10-week old infants, from the Cape Town region of South Africa were enrolled. All infants were routinely vaccinated with intradermal BCG (Statens Serum Institute, Copenhagen) within 48 hours of birth. Infants born to HIV-positive mothers, infants known to be HIV positive, infants with suspected or confirmed TB disease, and infants with any other active or chronic illnesses at the time of enrollment, were excluded. Human participation was according to the US Department of Health and Human Services and good clinical practice guidelines. This included protocol approval by the University of Cape Town research ethics committee and the UMDNJ Institutional Review Board (IRB). Written informed consent was obtained from all mothers whose babies took part in the study. Up to 10 ml whole blood was collected from each healthy infant.

### PBMC isolation, incubation and RNA purification

PBMC were isolated from peripheral venous blood by density gradient centrifugation, and cryopreserved. Later, PBMC were thawed, washed in RPMI 1640 (Biowhittaker, Walkersville, MD, USA), and "rested" for 6 hours at 1 × 10^6 ^cells in 1 ml 10% pooled human AB serum in RPMI 1640 supplemented with L-glutamine at 2 mM (Biowhittaker), at 37°C and in 5% CO_2_. PPD (20 μg/ml) or BCG, reconstituted from the vaccine vial as previously described [12], at an MOI of 0.18, was then added to the cells. PBMC incubated with medium alone served as negative (unstimulated) control. Incubation was continued for 12 hours at 37°C and in 5% CO_2_, after which the cells were harvested and processed with the QIAamp RNA Blood Mini Kit (Qiagen, Hilden, Germany), according to the manufacturer's instructions, to isolate RNA. RNA was DNAse treated using the on-column DNAse digestion kit (Qiagen, Hilden, Germany). A median of 0.37 μg (0.19–0.52 μg) RNA was obtained from 1 × 10^6 ^PBMC. The dose of BCG and the duration of incubation were found optimal in pilot experiments, which assessed up-regulation of multiple T-helper type 1 (Th1) cytokine, apoptosis and regulatory genes by real time RT-PCR (data not shown). The RNA was cryopreserved at -80°C for later use in DNA micro-array and RT-PCR experiments.

### RNA amplification protocol

A median 124 ng (range 63–174 ng) extracted RNA was amplified using the Illumina RNA Amplification Kit (Ambion, Austin, TX, USA), based on the Eberwine protocol [13]. A Biotin-16-UTP label was incorporated into amplified RNA during the *in vitro *transcription process (Perkin Elmer Life and Analytical Sciences, Woodbridge, Ontario, Canada). Amplification gave yields ranging from 1 μg to 25 μg. Amplified RNA (1000 ng per array) was hybridized to the Illumina HumanRefSeq-8 BeadChip according to the manufacturer's instructions (Illumina, San Diego, CA, USA). The HumanRefSeq-8 bead chip comprises of 24,000 sequences representing 16,238 genes from the curated portion of the NIH Reference Sequence Database . Each sequence is represented at least 30 times on the array. Arrays were scanned with an Illumina bead array reader confocal scanner, according to the manufacturer's instructions. Array data processing and analysis was performed using Illumina BeadStudio software.

### Real-Time RT-PCR

A median 124 ng (range 63–174 ng) RNA was reverse transcribed to cDNA using oligo-dT and the Omniscript Kit (Qiagen). cDNA was stored at -20°C until use. Real time PCR was performed using the Roche LightCycler^® ^and Quantitect mastermix (Qiagen).

Quantified, purified and diluted PCR product was used to generate external standard curves for each primer pair. Cycle number values were converted to copy number using these curves post amplification. All primers (synthesized by Integrated DNA Technologies, Toronto, Canada) were designed to span intron-exon sequences to distinguish between mRNA and genomic DNA (Additional file 1). 1 μl cDNA was used in each reaction. Cycling conditions consisted of an initial activation step of 15 minute at 95°C followed by 45 cycles of 15 seconds at 94°C, 20 seconds at 60°C and 15 seconds at 72°C, were used for each primer pair. To control for variation in cDNA quantity between samples the copy number of the gene of interest was divided by the copy number of the house keeping gene HPRT. All PCR reactions were performed in duplicate.

### Data analysis

Data was normalized using the VSN2 software "Variance stabilization and calibration for microarray data", which is available as part of the BioConductor project (an open source software project to provide tools for the analysis of genomic data) [14] Data files are available at the Gene Expression Omnibus data repository: GSE14408. Prior to the identification of differentially expressed genes, K-means cluster analysis (kmeans software, BioConductor) was performed to identify outlier samples. Unstimulated samples were found to cluster together and stimulated samples clustered together (with little differentiation between BCG and PPD), with no outliers, therefore all samples were included in subsequent analysis.

Differentially expressed genes were identified using eBayes which is a component of the Limma software package: Linear Models for Microarray data (BioConductor) [15]. eBayes is an empirical Bayes model which estimates the true expression signal by borrowing information across genes and increasing the stability of the analysis [16]. The statistics for differential expression provided by eBayes include the (log) fold change, moderated *t-*statistic (same as t-statistic except that the standard errors have been moderated across genes), *p*-value (based on moderated t-statistic), adjusted *p*-value (false discovery rate adjusted *p*-value) and *B*-statistic (log-odds that the gene is differentially expressed).

The expression level of genes differentially expressed by BCG stimulation (with an adjusted *p*-value <0.01) were compared to genes differentially expressed by PPD stimulation (with an adjusted *p*-value <0.01) using a 2-tailed Spearmans correlation (SPSS).

A sub-set of genes with an adjusted *p*-value of <0.01 and a >2 fold differential expression in PPD and BCG stimulated PBMC were selected for confirmation of expression by real-time RT-PCR. Differentially expressed genes were also assessed according to Gene Ontology (GO) categories (Onto-Express) and pathway analysis was performed using Pathway-Express [17].

## Results

### Genes up or down-regulated following incubation of PBMC with BCG or PPD

**Figure 1:**
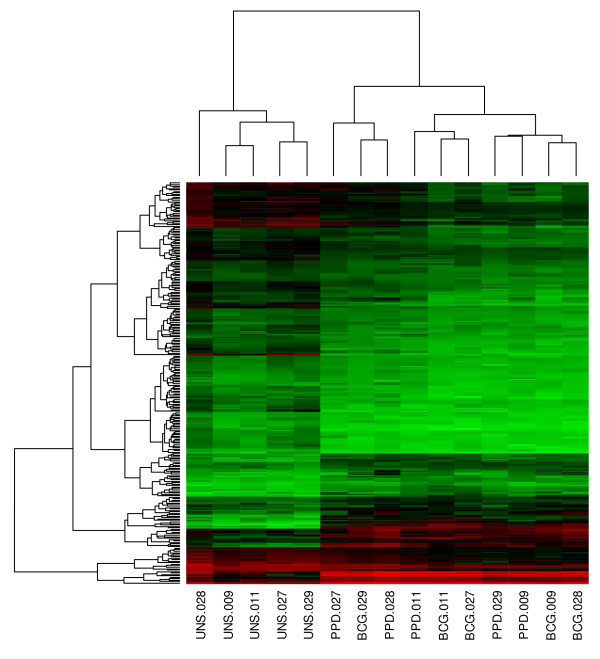
**Heatmap of gene expression profiles with a >2 fold change in expression in response to BCG stimulation, compared with the unstimulated control**. Genes were ordered according to their cluster determined by the k-means algorithm. Values increase from green to red, via black. The incubation conditions and the participant identifiers are given below the heatmap, e.g., "UNS.028" refers to PBMC incubated without any specific antigens (unstimulated) in participant 28.

Five 10-week old infants, routinely vaccinated with BCG at birth, were enrolled. DNA micro-array analysis was performed using amplified RNA, purified from cryopreserved PBMC that were later thawed and incubated with BCG, PPD or medium only (unstimulated) for 12 hours. Using K-means clustering, by the 3 incubation conditions and using all genes, unstimulated samples clustered away from BCG and PPD stimulated samples (Figure 1). However, BCG and PPD stimulated samples were not separated by this analysis (Figure 1). Overall, BCG induced differential expression of 411 genes (p < 0.01, eBayes), compared with the unstimulated control. Of the 411 genes, 136 were upregulated and 275 downregulated (Additional file 2). PPD induced differential expression in 291 genes (p < 0.01, eBayes, Additional file 2). Of the 291 genes, 95 were upregulated and 196 were downregulated. Of the significantly differentially expressed genes, 74 and 73 genes were up-regulated >2-fold by BCG and PPD, respectively; 201 and 127 genes were down-regulated >2-fold by these respective conditions (Additional file 2). We concluded that gene expression profiles obtained by stimulating PBMC with BCG were not sufficiently different from expression profiles obtained by stimulating cells with PPD to enable the samples to fall into separate clusters.

### Detailed comparison of expression profiles induced by BCG and PPD

Under both stimulation conditions interleukin 6 (IL-6), granulocyte monocyte colony stimulating factor (GM-CSF) and interleukin 1 family member 8 (IL1F9) showed the greatest increase in expression. Genes with the greatest decrease in expression, under both conditions, include fatty acid binding protein 4 (FABP4), colony stimulating factor 1 (CSF1)-receptor, and transforming growth factor beta 1 (TGF-β1) (Additional file 2).

**Figure 2:**
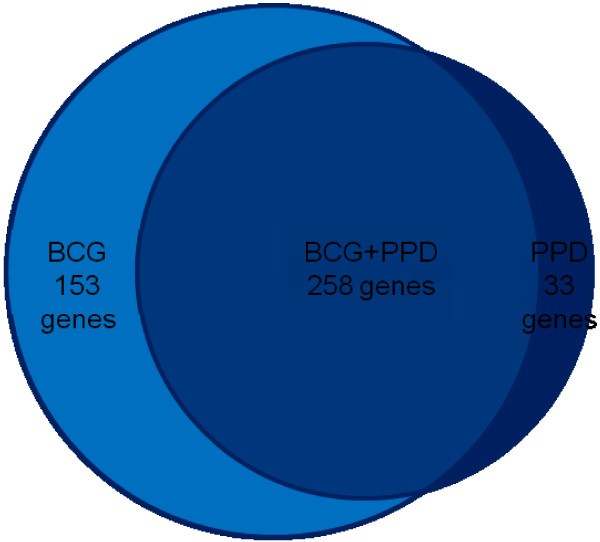
**Proportional Venn distribution of genes with a p-value <0.01 expressed in response to either BCG or PPD stimulation**. The Venn diagram shows the overlap in gene expression between the 411 genes induced by BCG and 291 genes induced by PPD stimulation.

**Figure 3:**
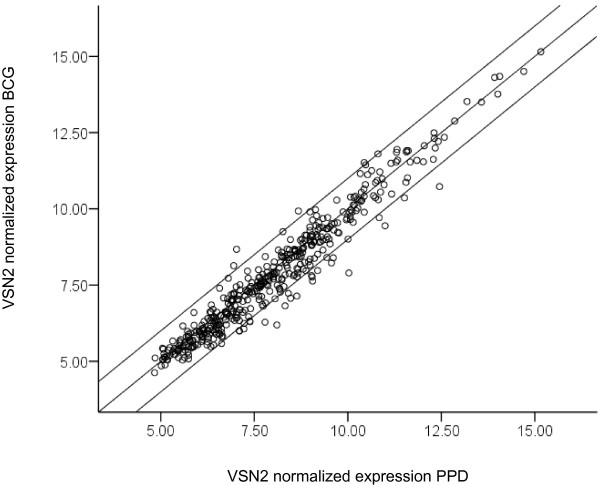
**Comparison of gene expression induced in response to stimulation with PPD and with BCG**. Data was normalized using a variance stabilization method (VSN2) and genes significantly induced by stimulation with PPD and BCG, as compared with the unstimulated control, were combined into a single gene list: the scatter of these genes in response to either stimulation is shown. Spearman's rank test was used to assess the correlation (r = 0.965, *p *< 0.000001). The lines indicate a greater than 2 fold difference in expression.

Next, we compared differential gene expression following BCG stimulation (411 genes) and PPD stimulation (291 genes) relative to un-stimulated controls. A large proportion of genes were expressed under both stimulation conditions with only 33 genes expressed in response to PPD only (Figure 2 and Additional file 3). The majority of genes were expressed to an equivalent degree, and a strong correlation between PPD and BCG stimulation was seen (r = 0.965, *P *< 0.000001; Figure 3). Only 29 genes were differentially expressed >2-fold between the two stimulation conditions (Additional file 4). BCG induced a >2 fold change in the expression of granulocyte colony stimulating factor (G-CSF), matrix metalloproteinase 1 (MMP1), guanylate binding protein 1(GBP1) and GM-CSF, compared with PPD. In turn, PPD induced a >2 fold increase in expression in chemokine (CXC motif) ligand 5 (CXCL5), thrombomodulin (THBD), cytokine-like nuclear factor n-pac (N-PAC) and thrombospondin 1 (THBS1), compared with BCG. A greater difference was seen in the number of genes down-regulated by BCG and PPD. BCG reduced the expression of 21 genes >2 fold when compared with PPD (Additional file 4).

We concluded that the majority of genes up-regulated or down-regulated by BCG stimulation were also up-regulated or down-regulated by PPD stimulation, when compared with un-stimulated controls.

### Confirmation of expression patterns with real-time RT-PCR

**Figure 4:**
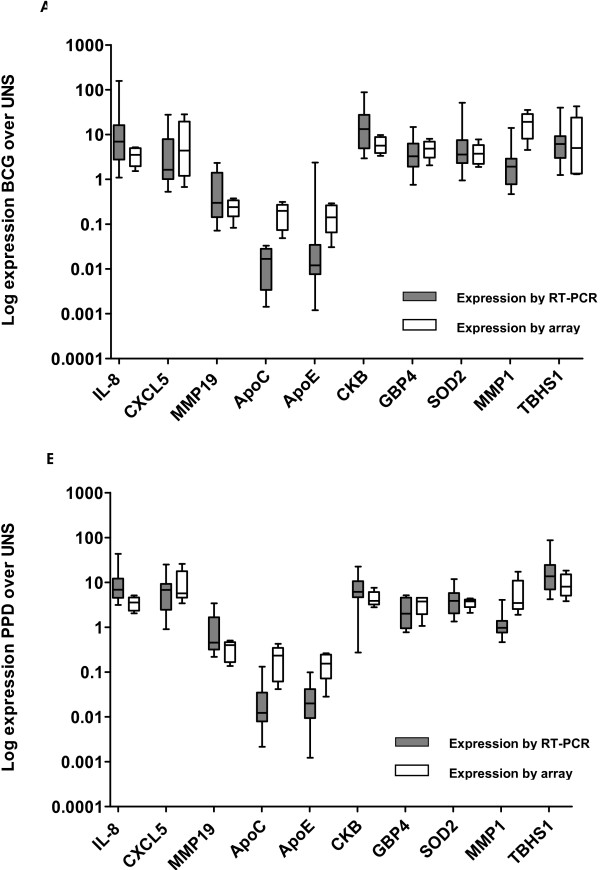
**Comparison of expression of highly and moderately expressed genes using DNA microarray and real time RT-PCR**. Gene expression shown is that in relation to media, for BCG (A), and PPD (B). Microarray analysis involved 5 infants, whereas real time RT-PCR analysis involved these 5 infants plus an additional 10 infants (total n = 15). Box plots represent median plus 95^th^, 75^th^, 50^th ^and 25^th ^percentiles.

To confirm the expression patterns of genes identified using the array, real time RT-PCR for specific genes was performed using total RNA from the 5 infants used for array analysis, and using RNA from 10 additional 10-week old infants, routinely vaccinated with BCG at birth (total of 15 infants for RT-PCR analysis). PBMC from the latter infants were processed identically to PBMC from infants whose RNA was examined in the array study. Primers were designed against selected transcripts and the copy number of each gene relative to the housekeeping gene HPRT was determined. There was concordance between DNA microarray and real-time RT-PCR results regarding expression of both highly and moderately expressed genes in response to both BCG (Figure 4A) and PPD (Figure 4B). When the median array expression from the 5 infants selected for array analysis was compared with the median RT-PCR expression from the total cohort of 15 infants, a significant correlation was found following both PPD (r = 0.806, *p *= 0.005, Spearman's test) and BCG stimulation (r = 0.697, *p *= 0.025, Spearman's test). Although the magnitude of the response was greater to BCG than PPD, the variability of response to stimulation with BCG was also greater.

We concluded that the patterns of gene expression detected by array analysis could be confirmed by real-time RT-PCR analysis.

### Ontology of genes differentially expressed in response to stimulation with BCG

**Figure 5:**
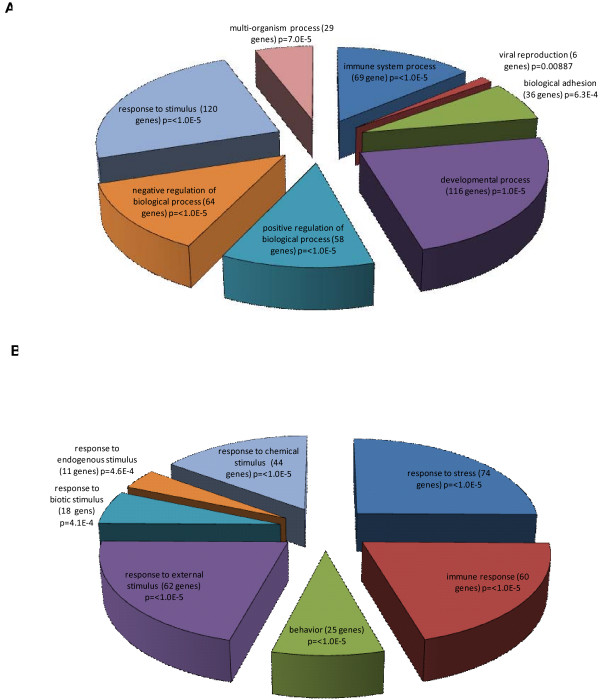
**Gene ontology analysis of genes differentially expressed in response to stimulation with BCG, as compared with the unstimulated control (n = 411)**. (A) Gene ontology terms for Response to stimulus parent term. (B) Gene ontology terms for the Response to external stimulus parent term.

Transcripts differentially expressed in response to stimulation with both BCG and PPD were analysed using the Gene Ontology (GO) tool Onto-Express an analysis tool available in the Onto-Tools ensemble [18]. As there was little differential gene expression between the two stimulation conditions the GO results obtained for BCG and PPD were highly similar (Additional file 5). Therefore, only GO results for the 411 genes expressed in response to BCG are shown in Figure 5.

The GO database allows for the classification of a gene based on its function, i.e., biological, cellular or molecular function. The greatest proportion of genes mapped to GO: Response to external stimulus (120 genes), *p *= < 1.0 × 10^-5 ^(Figure 5A). Sub-mapping revealed a strong statistical significance to GO: Response to stress (74 genes), *p *= < 1.0 × 10^-5 ^(Figure 5B) which comprises of a large number of genes in the inflammatory immune response (Additional file 5).

### Pathway analysis of differentially expressed genes

Pathway-Express (PE) is a pathway analysis tool in the Onto-Tools ensemble utilizing the available Kyoto Encyclopedia of Genes and Genomes (KEGG) pathway information [17,19]. This tool generates, firstly, a probability (*p) *value based on the number of genes present in a particular pathway. Secondly, a perturbation factor (gamma *p*) is calculated taking into consideration the (i) normalized fold change of the gene and (ii) the number of genes upstream of its position on a pathway based. The *p *value and gamma *p *value are then combined to obtain an impact factor which can be used to rank pathways in order of biological relevance. Following BCG stimulation, 21 pathways had a statistically significant gamma *p *value <0.01 and 17 pathways had a gamma *p *value <0.01 following PPD stimulation (Table 1). The pathway with the highest impact factor following BCG stimulation was Cell Adhesion Molecules (CAMs) with 4 genes down-regulated following stimulation with BCG (Additional file 6). The pathway with the highest impact factor following stimulation with PPD was the Hematopoietic cell lineage pathway (Additional file 6). Four pathways were significant following BCG stimulation, but not following PPD (Table 1).

**Table 1:** Pathway level analysis of genes differentially expressed following stimulation with BCG.

	**BCG stimulation**					**PPD stimulation**				
**Pathway Name**	**Rank**	**^a^Impact Factor**	**^b^#Genes in Pathway**	**^c^*p*-value**	**^d^gamma *p*-value**	**Rank**	**^a^Impact Factor**	**^b^#Genes in Pathway**	**^c^*p*-value**	**^d^gamma *p*-value**

Cell adhesion molecules (CAMs)	1	45.4	4/133	0.153	9.00E-19	-	-	-	-	-

Cytokine-cytokine receptor interaction	2	34.6	34/259	1.31E-12	3.51E-14	15	7.2	27/259	-5.50E-14	0.006

Hematopoietic cell lineage	3	30.6	14/88	8.34E-11	1.57E-12	1	27.3	12/88	2.68E-10	3.86E-11

Toll-like receptor signaling pathway	4	26.4	14/102	6.39E-10	9.56E-11	4	18.3	9/102	2.11E-06	2.14E-07

Fc epsilon RI signaling pathway	5	21.6	2/77	0.336	9.24E-09	11	10.2	2/77	0.211	4.24E-04

Jak-STAT signaling pathway	6	21.3	13/153	8.33E-07	1.22E-08	6	14.0	8/153	3.24E-04	1.21E-05

Graft-versus-host disease	7	20.1	5/42	4.65E-04	4.03E-08	3	18.6	5/42	9.93E-05	1.67E-07

MAPK signaling pathway	8	17.0	13/265	2.78E-04	7.67E-07	5	16.0	9/265	0.003	1.98E-06

TGF-beta signaling pathway	9	16.9	1/89	0.752	8.29E-07	10	10.6	3/89	0.079	3.01E-04

GnRH signaling pathway	10	15.8	1/97	0.781	2.29E-06	2	26.2	1/97	0.662	1.15E-10

Apoptosis	11	14.1	6/84	0.002	1.13E-05	7	13.4	4/84	0.014	2.24E-05

Type I diabetes mellitus	12	13.9	6/44	5.76E-05	1.33E-05	8	12.9	5/44	1.25E-04	3.43E-05

T cell receptor signaling pathway	13	13.1	6/93	0.003	2.84E-05	13	8.8	3/93	0.085	0.001

PPAR signaling pathway	14	12.5	8/69	1.14E-05	5.14E-05	9	12.0	7/69	1.16E-05	8.11E-05

Natural killer cell mediated cytotoxicity	15	11.4	5/131	0.054	1.34E-04	14	7.9	3/131	0.179	0.003

Type II diabetes mellitus	16	9.0	3/44	0.031	0.001	17	6.6	1/44	0.388	0.009

Adipocytokine signaling pathway	17	9.0	4/72	0.026	0.001	-	-	-	-	-

Epithelial cell signaling in Helicobacter pylori infection	18	8.6	4/69	0.022	0.002	12	9.5	3/69	0.041	7.85E-04

Complement and coagulation cascades	19	7.6	6/69	7.06E-04	0.004	-	-	-	-	-

Bladder cancer	20	6.9	3/42	0.027	0.008	16	6.9	3/42	0.011	0.008

Acute myeloid leukemia	21	6.8	5/57	0.002	0.008	-	-	-	-	-

^a ^calculated on basis of proportion of differentially expressed genes in a pathway and gene perturbation factors of all genes in the pathway

^b ^number of genes in a particular pathway

^c ^P value for pathways that contain a proportion of differentially expressed genes that is significantly different from what is expected by chance

^d ^This perturbation factor takes into consideration the (i) normalized fold change of the gene and (ii) the number of genes upstream of its position on a pathway

We concluded that BCG stimulation down-regulated the expression of proteins important for cell adhesion, and that pathway analysis is a useful tool for analyzing expression profiles induced by mycobacterial antigens.

### Expression of macrophage related genes

Mycobacteria are intracellular pathogens that primarily infect macrophages. The macrophage (Mφ) response to mycobacterial infection may be crucial in determining whether an infection is cleared or whether infection progresses to disease. Macrophages comprise a heterogeneous population of cells with diverse functions. Mφ1 support a protective Th1 response whereas Mφ2 display poor antigen-presentation capacity and suppress Th1 function [20-23]. We found that many genes up-regulated by both BCG and PPD in our infants' PBMC have previously been associated with adult Mφ1 macrophages, including IL-1β, IL-8, IL-6, tumour necrosis factor alpha (TNF-α), interferon-inducible protein 10 (IP10), macrophage inflammatory protein (MIP)-1β and macrophage derived chemokine (MDC) (Table 2) [4,21,24-26]. By analysing genes down-regulated by mycobacterial antigens, we revealed further evidence of macrophage polarization: genes in the peroxisome proliferator-activated receptor (PPAR) signaling pathway, associated with the polarization of macrophages into a Mφ2 phenotype, were down-regulated following stimulation with PPD and BCG, (Additional files 2 and 3) [22,23]. BCG stimulation down-regulated CD36, PPAR-γ, and retinoid × receptor (RXR) in the PPAR signaling pathway, presumably skewing monocytes towards the development of a Mφ1 phenotype.

**Table 2:** Genes identified as up-regulated response to antigen stimulation in previous studies with mycobacterial antigens.

**Genbank**	**Symbol**	*Fold PPD	*Fold BCG	*BCG over PPD	**Cell type**	**Stimulus**	**Reference**
NM_000600	IL-6	67.97	83.42	1.23	Mφ1, Mφ, DC	M.tb, BCG	Chaussabel *et. al*. 2003 Verrek *et. al*. 2006 Begum *et. al*. 2004

NM_000576	IL-1 β	17.34	15.10	0.87	Mφ1, Mφ, DC, Guinea-pig splenocytes	M.tb, BCG, PPD	Chaussabel *et. al*. 2003 Verrek *et. al*. 2006 Begum *et. al*. 2004 Khajoee *et. al*. 2006 Tree *et. al*. 2006

NM_001511	GRO-1, CXCL1	27.12	16.89	0.62	Mφ1, Mφ, DC	M.tb, BCG	Chaussabel *et. al*. 2003 Khajoee *et. al*. 2006

NM_002421	MMP1	7.21	17.18	2.38	Mφ1, Mφ, DC	M.tb, BCG	Chaussabel *et. al*. 2003

NM_002089	GRO-2, CXCL2, MIP-2A	19.24	24.10	1.25	Mφ1, Mφ, DC	M.tb	Chaussabel *et. al*. 2003

NM_002090	GRO-3, CXCL3	15.94	9.91	0.62	Mφ1	M.tb	Chaussabel *et. al*. 2003

NM_000594	TNF-α	4.86	9.49	1.95	Mφ1, Mφ, DC	M.tb, BCG	Chaussabel *et. al*. 2003 Verrek *et. al*. 2006 Begum *et. al*. 2004

NM_000963	PTGS2, COX2	10.35	13.17	1.27	Mφ, DC	M.tb	Chaussabel *et. al*. 2003

NM_002984	MIP-1β, CCL4	4.19	5.47	1.31	Mφ1, Mφ, DC	M.tb	Chaussabel *et. al*. 2003 Verrek *et. al*. 2006 Cliff *et al*. 2004

NM_007115	TNFAIP6.	9.10	7.12	0.78	Mφ, DC	M.tb, BCG	Chaussabel *et. al*. 2003 Khajoee *et. al*. 2006

NM_000584	IL-8	3.62	3.60	0.99	Mφ1, Mφ, DC, Guinea-pig splenocytes, CD4+	M.tb, BCG, PPD	Chaussabel *et. al*. 2003 Verrek *et. al*. 2006 Khajoee *et. al*. 2006 Tree *et. al*. 2006 Cliff *et al*. 2004

NM_139266	STAT-1	2.41	4.52	1.88	Mφ, DC Mφ	M.tb PPD	Chaussabel *et. al*. 2003, Begum *et. al*. 2004

NM_015714	GOS-2	4.36	4.26	0.98	Mφ, DC	M.tb	Chaussabel *et. al*. 2003

NM_004184	WARS	1.52	2.29	1.51	Mφ, DC Guinea-pig splenocytes	M.tb PPD	Chaussabel *et. al*. 2003 Tree *et. al*. 2006

NM_000595	LTA	2.31	2.28	0.99	Mφ, DC	M.tb	Chaussabel *et. al*. 2003

NM_001955	EDN1	1.93	3.41	1.77	Mφ, DC	M.tb	Chaussabel *et. al*. 2003

NM_001565	IP-10, CXCL10	2.28	3.56	1.56	Mφ, DC	M.tb	Chaussabel *et. al*. 2003

NM_002462	MX1	1.26	1.59	1.26	Mφ, DC	M.tb	Chaussabel *et. al*. 2003

NM_002164	INDO	3.01	2.42	0.80	Mφ, DC		Chaussabel *et. al*. 2003

NM_002460	IRF4	1.56	1.89	1.21	Mφ, DC	M.tb	Chaussabel *et. al*. 2003

NM_002990	MDC, CCL22	3.53	3.12	0.88	Mφ1	M.tb	Verrek *et. al*. 2006

NM_005746	PBEF	3.36	2.37	0.71	Mφ	BCG	Begum *et. al*. 2004

NM_002187	IL-12p40	1.98	2.66	1.35	Mφ	BCG	Begum *et. al*. 2004

NM_004591	CCL20, MIP3A	24.49	37.96	1.55	Mφ2	BCG	Khajoee *et. al*. 2006

NM_000636	SOD2	4.25	3.61	0.85	Mφ1	BCG	Khajoee *et. al*. 2006

NM_004049	BCL2A1	2.75	2.55	0.93	Mφ1 CD8+	BCG, M.tb	Khajoee *et. al*. 2006 Cliff *et. al*. 2004

NM_002053	GBP1	2.31	5.25	2.28	Bovine PBMC	PPD	Meade *et. al*. 2006

NM_000758	GM-CSF	36.79	77.65	2.11	Guinea-pig splenocytes	PPD	Tree *et. al*. 2006

NM_000575	IL-1alpha	21.60	22.38	1.04	Guinea-pig splenocytes	PPD	Tree *et. al*. 2006

NM_003745	SOCS-1	1.65	2.37	1.44	CD8+	M.tb	Cliff *et al*. 2004

NM_172219	CSF3, G-CSF	2.93	9.17	3.13	CD8+	M.tb	Cliff *et al*. 2004

Genes reported as differentially expressed in previous studies and also differentially expressed in this current study are listed. * Median fold change in normalised expression.

We concluded that PPD and BCG stimulation of PBMC results in gene modulation that supports development of a Mφ1 phenotype.

## Discussion

BCG is likely to remain the cornerstone of future TB vaccination strategies. We therefore used microarray analysis to determine gene expression profiles of BCG vaccinated infant PBMC following ex vivo stimulation with BCG or PPD. Overall, we demonstrated a remarkably similar expression profile following stimulation with the two reagents. Our study, the first investigation of mycobacteria-induced gene expression in such a young vaccinated infant population, shows that a greater number of genes were down-regulated in response to ex vivo stimulation with either antigen, compared with up-regulated genes. In the absence of a better biomarker the measurement of IFN-γ protein following stimulation with mycobacterial antigen remains the assay of choice for the assessment of TB vaccine "take" and TB vaccine efficacy [27]. In our study IFN-γ mRNA was up-regulated 2.12 fold in response to PPD stimulation and 2.19 for BCG stimulation but did not reach an adjusted p-value of <0.01. It was somewhat surprising that we did not see a stronger induction of IFN-γ mRNA in response to PPD and BCG stimulation. However, we have consistently observed in our studies that the fold increase in antigen specific IFN-γ mRNA is lower than that of IFN-γ protein. This is most likely due to the basal level of IFN-γ mRNA expression masking the increase in IFN-γ mRNA production by antigen specific cells. Others have also found that IFN-γ protein expression is not always directly correlated with IFN-γ mRNA expression [28]. IFN-γ can be controlled at the transcriptional, post-transcriptional and translational levels which may also account for the discrepancy in IFN-γ protein and mRNA production [29,30].

In our study, for both BCG and PPD, genes with the greatest increase in expression included IL-6, GM-CSF and IL1F9. IL-6 and GM-CSF are well-known proinflammatory proteins, and IL1F9, a member of the pro-inflammatory IL-1 family of proteins binds to the receptor IL-1 receptor related protein 2 (IL-1Rrp2) and induces nuclear factor kappa B (NFκB) [31]. NFκB is a transcription factor that acts as a master regulator of the proinflammatory immune response, orchestrating induction of many type 1 cytokines, including IL-6 and IL-8.

Genes with the greatest decrease in expression included FABP4, M-CSF receptor, GSN and TGF-β1. FABP is strongly upregulated in human bronchial epithelial by the Th2 cytokines IL-4 and IL-13, and is down-regulated by IFN-γ [32]. We postulate that the down-regulation of FABP4 in our study may be due to the induction of IFN-γ protein by PPD and BCG stimulation. As regards M-CSF, monocytes infected with mycobacteria and cultured with this cytokine produce high levels of IL-10 and fail to stimulate T cells [33]. Down-regulation of the M-CSF receptor may prevent the M-CSF driven differentiation of monocytes into IL-10 producing cells. GSN is required for rapid motile responses in cells involved in inflammation, and wound healing and down-regulation of this gene may lead to suppression of inflammatory immune responses [34]. As TGF-β1 is a key cytokine in the maintenance of tolerance [35-37], TGF-β1 down-regulation may result in a lesser immune suppression of effector T cell responses.

We had expected to find large differences between the responses to ex vivo stimulation with BCG and PPD, given that BCG is a live bacterium and PPD is a mixture of secreted proteins from *M.tb*. However, few genes were differentially expressed >2 fold in response to stimulation with BCG, when compared with PPD. Stimulation with BCG induced >2 fold more G-CSF and GM-CSF, both cytokines involved in the activation and differentiation of monocytes into macrophages. Stimulation with PPD induced higher levels of thrombomodulin (THBD) and thrompospondin (THBS1), both involved in immune regulation [38,39]; for example, mice with a mutation in the THBD gene have uncontrolled lung inflammation in response to mycobacterial infection [39]. Overall, the magnitude of response to BCG stimulation was greater than that seen with PPD, which may be due to differences in antigen composition and concentration or may be a reflection of increased activation of toll like receptors by the lipid components of the BCG cell wall. Pathway analysis reveals a decrease in the expression of cell adhesion molecules (CAMs) in response to ex vivo BCG, but not to ex vivo PPD stimulation. The genes in this pathway include CD86, activated leukocyte cell adhesion molecule (ALCAM), ITGAM and claudin 1 (CLDN1). CD86 is a costimulatory molecule, important for T cell activation. ALCAM interaction with CD6, present on mature T cells, is important for T cell proliferation [40]. Downregulation of CD86 and ALCAM may therefore represent immunosuppressive mechanisms employed by infecting mycobacteria. ITGAM is a subunit of the complement receptor CR3, is expressed on monocytes and macrophages and is one of multiple receptors used by mycobacteria to gain entry into the macrophage [41]. Down-regulation of ITGAM may therefore help protect cells from infection with mycobacteria. The cell adhesion molecule CLDN1 has recently been described as a receptor for hepatitis C virus entry into the cell [42]; its role in mycobacteral pathogenesis is unclear. It is possible that differences in innate responses to BCG versus PPD would have been noted if the PBMC tested had been obtained prior to BCG vaccination and induction of an acquired mycobacteria specific T cell response. However, in this study as infants were vaccinated within 48 hours of birth we were not able to collect a blood sample prior to BCG vaccination.

The PPAR signaling pathway, associated with the development of alternative (Mφ2) macrophages, was down-regulated by both BCG and PPD. Genes associated with Mφ1 cells were up-regulated, suggesting a strong bias towards the development of Mφ1 cells in infants vaccinated with BCG. Mφ1 and Mφ2 macrophages can be generated in vitro through the culture of CD14+ positive cells with GM-CSF and M-CSF, respectively. We found strong up-regulation of GM-CSF and down-regulation of the M-CSF receptor in PBMC stimulated with BCG and PPD. These findings strongly suggest induction of conditions favorable for the development of protective Mφ1, rather than inhibitory Mφ2.

Many of the infant PBMC genes up-regulated in our study have also been recognized as up-regulated in response to mycobacterial antigen stimulation in previous studies of adult macrophages [4,21,24,26], adult dendritic cells [4], and CD4 and CD8 T cells [25], as well as guinea pig splenocytes [43] and bovine PBMC [11] (Table 2). There was a particularly striking similarity between the gene lists obtained from our BCG vaccinated infants and those generated by Chaussabel, *et al*., who incubated *M.tb *with monocytes and dendritic cells [4]. Wu, *et al*. used real time RT-PCR to study 17 immune genes in infants vaccinated with different strains of BCG at birth and found that Danish BCG induced IFN-γ, IL-12β and IL-27 [44]. Danish BCG was also used to vaccinate the infants in our study and we saw a modest (~2 fold) increase in IFN-γ and IL-12β, but not in IL-27, in response to stimulation with BCG and PPD. The difference in our results may be due to the time point at which PBMC were isolated: 10 weeks post-BCG vaccination in our study versus 12 months after vaccination by Wu, *et al*. In addition, we restimulated with BCG or with PPD, whereas Wu, *et al*. used culture filtrate proteins of *M. tb *as antigen in their assays.

## Conclusion

The early identification of promising vaccine candidates in phase I and II clinical trials is essential if we are to expedite the development of new TB vaccines and use precious resources most effectively. The first wave of new TB vaccines to reach clinical trials have been designed as booster vaccines for BCG and initial phase II efficacy trials with these vaccines will be conducted in BCG vaccinated populations prior to TB exposure. Infants in Africa are a good target population for such efficacy trials as they will have been recently vaccinated with BCG. Infants are less likely to have had exposure to TB, compared with older children or adults. Understanding base-line responses to BCG vaccination will aid the design of new vaccine strategies. We have successfully used microarray analysis to study immune responses of healthy infants vaccinated with BCG at birth. Analysis of expression profiles, in particular down-regulated genes, has given fresh insight into pathways activated by ex vivo exposure to BCG and PPD that add to our knowledge of the infant immune response to BCG vaccination.

## Authors' contributions

HF participated in the study design, carried out the microarray studies and data analysis and drafted the manuscript. AK participated in the study design and carried out the microarray studies and real time RT-PCR. MB helped with data analysis and helped to draft the manuscript. GH arranged the ethical permissions and collection of clinical samples. PS, GK and AVSH participated in the study design and helped to draft the manuscript. WH participated in the study design, directed the data analysis and helped draft the manuscript. All authors read and approved the final manuscript.

## Pre-publication history

The pre-publication history for this paper can be accessed here:



## Supplementary Material

Additional File 1 **Primers designed to confirm differential expression of genes identified using microarray analysis.** Details of primer sequences, primer annealing temperatures and PCR product sizes. Click here for file

Additional File 2 **BCG and PPD *p*-values.** Log fold change, *p*-values, adjusted *p*-values, moderated t-statistic and moderated B-statistic values for each gene differentially expressed between stimulated and unstimulated PBMC with an adjusted *p*-value < 0.01. Click here for file

Additional File 3 **BCGvPPD venn lists.** List of genes differentially expressed between BCG and PPD stimulation as determined by venn analysis. Click here for file

Additional File 4 **Genes with a greater than 2 fold difference in expression in response to stimulation with BCG when compared to PPD.** Annotation details of the genes differentially expressed between BCG and PPD stimulation. Click here for file

Additional File 5 **Genes in GO Categories.** Lists of genes that fall into the different GO categories with accession number and gene symbol. Click here for file

Additional File 6 **pathways express lists.** Lists of genes represented in the Kegg pathways (gene symbols). Click here for file
