# Supplementary material for: Transcriptional profiling of mycobacterial antigen-induced responses in infants vaccinated with BCG at birth
Source: BMC Med Genomics. 2009 Feb 24;2:10. doi: 10.1186/1755-8794-2-10 (PMC2654906; doi:10.1186/1755-8794-2-10)
Supplement: Additional File 1 — Primers designed to confirm differential expression of genes identified using microarray analysis. Details of primer sequences, primer annealing temperatures and PCR product sizes. [file 1755-8794-2-10-S1.doc]

Table 1. Primers designed to confirm differential expression of genes identified using microarray analysis.

| Gene | Accession number | Primer sequence | Bases | Tm | product size |
| --- | --- | --- | --- | --- | --- |
| HPRT | NM_000194.1 | F TAT GGA CAG GAC TGA ACG TC | 211-230 | 60 | 64 |
|  |  | R CTA CAA TGT GAT GGC CTC CC | 275-256 | 62 |  |
| APOC1 | NM_001645.3 | F GTGGTGGTTCTGTCGATCG | 121-139 | 60 | 164 |
|  |  | R CGCATCTTGGCAGAAAGTTC | 265-284 | 60 |  |
| APOE | NM_000041.2 | F GTCGCTTTTGGGATTACC | 229-248 | 60 | 126 |
|  |  | R CCTTCAACTCCTTCATGGTC | 335-354 | 60 |  |
| CKB | NM_001823.3 | F GTCCTACGAAGTGTTCAAGG | 320-339 | 60 | 148 |
|  |  | R CGAGCTCAGCACGTAGTTG | 449-467 | 60 |  |
| CXCL5 | NM_002994.3 | F GTCCTTCGAGCTCCTTGTG | 156-174 | 60 | 173 |
|  |  | R GGCGAACACTTGCAGATTAC | 309-328 | 60 |  |
| GBP4 | NM_052941.2 | F GAGGGAATCATTGTCACTGG | 938-957 | 60 | 95 |
|  |  | R GCATTCTCCAGACAAGGTAC | 1013-1032 | 60 |  |
| IL-8 | NM_000584.2 | F CTTCCTGATTTCTGCAGCTC | 137-156 | 60 | 138 |
|  |  | R CCACTCTCAATCACTCTCAG | 274-255 | 60 |  |
| MMP1 | NM_002421.2 | F CTGATGTGGCTCAGTTTGTC | 355-374 | 60 | 166 |
|  |  | R GTGAATGTCAGAGGTGTGAC | 501-520 | 60 |  |
| MMP19 | NM-2429.4 | F CAGGAAGCATCTGAACTTCC | 302-321 | 60 | 166 |
|  |  | R GGTTCAAGATGCGGAAAGTC | 448-467 | 60 |  |
| SOD2 | NM_000636.2 | F GTGAACAACCTGAACGTCAC | 329-348 | 60 | 101 |
|  |  | R CCATTGAACTTCAGTGCAGG | 410-429 | 60 |  |
| THBS1 | NM_003246.2 | F GATAGCCTCAACAACCGATG | 1398-1417 | 60 | 150 |
|  |  | R GATCACACCATCACCACATG | 1547-1528 | 60 |  |
