# Supplementary material for: Transcriptional profiling of mycobacterial antigen-induced responses in infants vaccinated with BCG at birth
Source: BMC Med Genomics. 2009 Feb 24;2:10. doi: 10.1186/1755-8794-2-10 (PMC2654906; doi:10.1186/1755-8794-2-10)
Supplement: Additional File 4 — Genes with a greater than 2 fold difference in expression in response to stimulation with BCG when compared to PPD. Annotation details of the genes differentially expressed between BCG and PPD stimulation. [file 1755-8794-2-10-S4.doc]

Table 3. Genes with a greater than 2 fold difference in expression in response to stimulation with BCG when compared to PPD

| **Genbank** | **Symbol** | **Definition** | **Go Process** | **BCG over PPD** |
| --- | --- | --- | --- | --- |
| NM_172219.1;NM_172220.1 | CSF3 | colony stimulating factor 3 (granulocyte) (CSF3), transcript variant 2, mRNA. Colony stimulating factor 3 (granulocyte) (CSF3), transcript variant 3, mRNA. | positive regulation of cell proliferation; cell surface receptor linked signal transduction; cellular defense response; development; cell-cell signaling | 3.13 |
| NM_002421.2 | MMP1 | matrix metalloproteinase 1 (interstitial collagenase) (MMP1), mRNA. | proteolysis and peptidolysis; collagen catabolism | 2.38 |
| NM_002053.1 | GBP1 | guanylate binding protein 1, interferon-inducible, 67kDa (GBP1), mRNA. | immune response | 2.28 |
| NM_000758.2 | CSF2 | colony stimulating factor 2 (granulocyte-macrophage) (CSF2), mRNA. | cell surface receptor linked signal transduction; cellular defense response; development | 2.11 |
| NM_003246.2 | THBS1 | thrombospondin 1 (THBS1), mRNA. | blood coagulation; cell motility; development; neurogenesis; cell adhesion | 0.43 |
| NM_032569 | N-PAC | cytokine-like nuclear factor n-pac (N-PAC), mRNA | pentose-phosphate shunt | 0.37 |
| NM_000361.2 | THBD | thrombomodulin (THBD), mRNA. | blood coagulation | 0.35 |
| NM_002994.3 | CXCL5 | chemokine (C-X-C motif) ligand 5 (CXCL5), mRNA. | positive regulation of cell proliferation; chemotaxis; signal transduction; immune response; cell-cell signaling; inflammatory response | 0.30 |
| NM_025201.3 | pp9099 | PH domain-containing protein (pp9099), mRNA. |  | 0.50 |
| NM_000399.2 | EGR2 | early growth response 2 (Krox-20 homolog, Drosophila) (EGR2), mRNA. | brain development; peripheral nervous system development; regulation of transcription, DNA-dependent; mechanosensory behavior | 0.49 |
| NM_032265.1 | ZMYND15 | zinc finger, MYND domain containing 15 (ZMYND15), mRNA. |  | 0.49 |
| NM_012212.2 | LTB4DH | leukotriene B4 12-hydroxydehydrogenase (LTB4DH), mRNA. | leukotriene metabolism | 0.48 |
| NM_015444.1 | RIS1 | Ras-induced senescence 1 (RIS1), mRNA. |  | 0.47 |
| NM_032717.3 | MGC11324 | hypothetical protein MGC11324 (MGC11324), mRNA. | metabolism | 0.47 |
| NM_005849.1 | IGSF6 | immunoglobulin superfamily, member 6 (IGSF6), mRNA. | cell surface receptor linked signal transduction; immune response | 0.46 |
| NM_080725.1 | C20orf139 | chromosome 20 open reading frame 139 (C20orf139), mRNA. |  | 0.46 |
| NM_000104.2 | CYP1B1 | cytochrome P450, family 1, subfamily B, polypeptide 1 (CYP1B1), mRNA. | eye morphogenesis (sensu Drosophila); electron transport; eye morphogenesis (sensu Mammalia) | 0.45 |
| NM_014397.3 | NEK6 | NIMA (never in mitosis gene a)-related kinase 6 (NEK6), mRNA. | protein amino acid phosphorylation | 0.45 |
| NM_015149.2 | RGL | RalGDS-like gene (RGL), mRNA. | small GTPase mediated signal transduction; neuropeptide signaling pathway | 0.44 |
| NM_022833.1 | DKFZP434H0820 | hypothetical protein DKFZp434H0820 (DKFZP434H0820), mRNA. |  | 0.43 |
| NM_022059.1 | CXCL16 | chemokine (C-X-C motif) ligand 16 (CXCL16), mRNA. | chemotaxis; immune response | 0.43 |
| NM_001295.1 | CCR1 | chemokine (C-C motif) receptor 1 (CCR1), mRNA. | chemotaxis; cell adhesion; invasive growth; cell surface receptor linked signal transduction; immune response; cell-cell signaling; inflammatory response | 0.41 |
| NM_000887.3 | ITGAX | integrin, alpha X (antigen CD11C (p150), alpha polypeptide) (ITGAX), mRNA. | histogenesis and organogenesis; cell adhesion; integrin-mediated signaling pathway; cell-matrix adhesion | 0.40 |
| NM_005572.2 | LMNA | lamin A/C (LMNA), transcript variant 2, mRNA. | muscle development; cell shape and cell size control | 0.38 |
| NM_002183.2 | IL3RA | interleukin 3 receptor, alpha (low affinity) (IL3RA), mRNA. | protein amino acid phosphorylation; development | 0.35 |
| NM_005211.2 | CSF1R | colony stimulating factor 1 receptor, formerly McDonough feline sarcoma viral (v-fms) oncogene homolog (CSF1R), mRNA. | signal transduction; development; cell proliferation; antimicrobial humoral response (sensu Invertebrata); transmembrane receptor protein tyrosine kinase signaling pathway; protein amino acid phosphorylation | 0.35 |
| NM_198125.1 | TYROBP | TYRO protein tyrosine kinase binding protein (TYROBP), transcript variant 2, mRNA. | cellular defense response; signal transduction; intracellular signaling cascade | 0.34 |
| NM_000784.2 | CYP27A1 | cytochrome P450, family 27, subfamily A, polypeptide 1 (CYP27A1), nuclear gene encoding mitochondrial protein, mRNA. | electron transport | 0.27 |
| NM_004994.1 | MMP9 | matrix metalloproteinase 9 (gelatinase B, 92kDa gelatinase, 92kDa type IV collagenase) (MMP9), mRNA. | proteolysis and peptidolysis; collagen catabolism | 0.23 |
